# Phosphate binding by sucroferric oxyhydroxide ameliorates renal injury in the remnant kidney model

**DOI:** 10.1038/s41598-018-38389-3

**Published:** 2019-02-11

**Authors:** Yoshikazu Nemoto, Takanori Kumagai, Kenichi Ishizawa, Yutaka Miura, Takeshi Shiraishi, Chikayuki Morimoto, Kazuhiro Sakai, Hiroki Omizo, Osamu Yamazaki, Yoshifuru Tamura, Yoshihide Fujigaki, Hiroshi Kawachi, Makoto Kuro-o, Shunya Uchida, Shigeru Shibata

**Affiliations:** 1 Division of Nephrology, Department of Internal Medicine, Teikyo University School of Medicine, Tokyo, Japan; 2 Division of Anti-aging Medicine, Center for Molecular Medicine, Jichi Medical University, Tokyo, Japan; 3 Department of Cell Biology, Kidney Research Center, Niigata University Graduate School of Medical and Dental Sciences, Niigata, Japan; 4 Division of Clinical Epigenetics, Research Center for Advanced Science and Technology, The University of Tokyo, Tokyo, Japan

## Abstract

Recent clinical studies indicate that the disturbed phosphate metabolism in chronic kidney disease (CKD) may facilitate kidney injury; nonetheless, the causal role of phosphate in CKD progression remains to be elucidated. Here, we show that intestinal phosphate binding by sucroferric oxyhydroxide (SF) ameliorates renal injury in the rat remnant kidney model. Sprague-Dawley rats received 5/6 nephrectomy (RK) and had a normal chow or the same diet containing SF (RK + SF). RK rats showed increased plasma FGF23 and phosphate levels, which were suppressed by SF administration. Of note, albuminuria in RK rats was significantly ameliorated by SF at both 4 and 8 weeks. SF also attenuated glomerulosclerosis and tubulointerstitial injury. Moreover, several different approaches confirmed the protective effects on podocytes, explaining the attenuation of glomerulosclerosis and albuminuria observed in this study. As a possible mechanism, we found that SF attenuated renal inflammation and fibrosis in RK rats. Interestingly, von Kossa staining of the kidney revealed calcium phosphate deposition in neither RK nor RK + SF rats; however, plasma levels of calciprotein particles were significantly reduced by SF. These data indicate that latent positive phosphate balance accelerates CKD progression from early stages, even when overt ectopic calcification is absent.

## Introduction

Chronic kidney disease (CKD) is a public health problem worldwide, contributing to deaths from end-stage renal disease and cardiovascular disorders^1,2^. Because the precise mechanisms for the CKD progression remain largely undetermined, the identification and intervention against major risk factors, including hypertension, proteinuria, and impaired glucose tolerance, are the mainstay to prevent the decline in kidney function. Several clinical studies indicate that phosphate overload may deteriorate kidney function^3^. Previously, we also reported that hyperphosphatemia is an independent risk factor for CKD progression^4^. Remarkably, higher plasma phosphate levels, even within the normal ranges, were associated with the decline in estimated glomerular filtration rate (GFR) in CKD patients^4^. However, the causal role of phosphate overload in CKD progression remains undetermined.

Mineral and bone disorders (MBD) are frequently associated with CKD. Reduced phosphate excretion from the kidney and its accumulation in the body stimulate the production of a phosphaturic hormone fibroblast growth factor 23 (FGF23) namely in osteocytes^5,6^. FGF23 then decreases phosphate reabsorption by inhibiting sodium-dependent phosphate transporters NaPi-2a and NaPi-2c^5,7,8^. FGF23 also reduces 1,25(OH)_2_ vitamin D levels by inducing the expression of 25-hydroxyvitamin D-24-hydroxylase, thereby decreasing intestinal phosphate absorption. Besides FGF23, parathyroid hormone (PTH) is also stimulated by high extracellular phosphate levels, which increases renal phosphate excretion by inhibiting NaPi transporters^6,9^. However, these mechanisms cannot fully compensate for the reduced renal phosphate excretion in advanced CKD, resulting in phosphate accumulation and hyperphosphatemia. In addition, secondary hyperparathyroidism aggravates hyperphosphatemia through excessive bone absorption^10^.

Previous studies indicate that high phosphate levels in the blood can significantly affect cardiovascular function^5,6,11^. Phosphate accumulation in advanced CKD patients results in extensive vascular calcification^11^, which plays a critical role in high cardiovascular mortality in these patients. Moreover, when the vascular smooth muscle cells are exposed to high extracellular phosphate, these cells transit to osteoblast-like cells and express osteogenic genes including Runx2, BMP-2, and Msx2^8,11,12^, further contributing to the progression of calcium deposition. Similarly, calcium phosphate deposition in the renal parenchyma can also deteriorate kidney function^11,13–16^. However, the injurious effects of phosphate in the kidney that are independent of ectopic calcification are not well characterized. In the plasma, mineral binding proteins such as fetuin-A sequesters a CaPi nanoparticle by binding and forming the complex with the mineral core (calciprotein particle; CPP)^8,17,18^. These particles primarily act to prevent the growth of CaPi crystals. Nonetheless, its accumulation in pathological conditions can induce pro-inflammatory responses and apoptotic pathways^8,19,20^. Given that plasma CPP levels increase with CKD progression^21,22^, it has been postulated that CPP may deteriorate kidney function in CKD subjects^8^.

Based on these observations, we here tested whether the latent positive phosphate balance at an early stage of the rat remnant kidney (RK) model can facilitate kidney damage. In this study, we show that the intervention against the disturbed phosphate metabolism by sucroferric oxyhydroxide (SF), a phosphate binder, ameliorates the progression of kidney dysfunction.

## Results

### Effects of sucroferric oxyhydroxide (SF) on phosphate metabolism in remnant kidney (RK) rats

**Figure 1:**
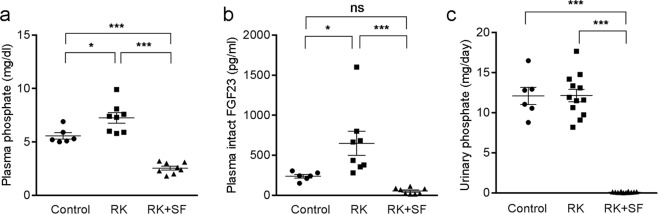
Phosphate and FGF-23 levels in control, remnant kidney rats (RK), and RK rats received sucroferric oxyhydroxide (RK + SF). (**a,b**) Plasma levels of phosphate (**a**) and intact FGF23 (**b**) in control, RK, RK + SF rats at 8 weeks (*n* = 6 for control, *n* = 8 for RK, *n* = 8 for RK + SF groups). (**c**) Urinary excretion of phosphate at 8 weeks in the indicated animals (*n* = 6 for control, *n* = 12 for RK, *n* = 12 for RK + SF groups). Data are expressed as mean ± SEM; *P < 0.05; ***P < 0.001. ns, not significant. n.d., not determined.

Male Sprague-Dawley rats received 5/6 nephrectomy and were randomly assigned to RK or RK with sucroferric oxyhydroxide (50 mg/g chow; RK + SF) group and were maintained for 8 weeks. Rats without 5/6 nephrectomy served as control. All the groups received normal diet. RK and RK + SF rats showed significant reduction in creatinine clearance (CCr) compared with control rats (Table 1). BUN was significantly increased in RK group compared with control rats; there were no significant difference in BUN levels between RK and RK + SF. Plasma phosphate levels were modestly but significantly elevated (Fig. 1a), whereas the levels of phosphaturic hormone FGF23 were highly increased in RK rats (Fig. 1b). Urinary phosphate levels were similar between control and RK rats (Fig. 1c). However, fractional excretion of phosphate was increased in RK rats (Table 1). In RK + SF rats, plasma phosphate and FGF-23 levels were significantly lower than RK rats (Fig. 1). SF administration also reduced urinary phosphate excretion, confirming the ability of SF to prevent phosphate absorption in the intestine. Levels of plasma calcium and 1,25(OH)_2_ vitamin D were significantly higher in RK + SF rats (Table 1), which can be explained by the suppression of FGF23. Blood pressure levels were similar between RK and RK + SF rats at baseline. Interestingly, however, blood pressure elevation was partially attenuated by SF at 8 weeks (but not at 4 weeks). Hemoglobin levels were not significantly different among three groups.

**Table 1:** Biological parameters at 0, 4, and 8 weeks.

Group	0 week			4 week			8 week		
	Control	RK	RK + SF	Control	RK	RK + SF	Control	RK	RK + SF
Systolic BP (mmHg)	100 ± 1	106 ± 2	107 ± 3	100 ± 2	126 ± 3^a^	127 ± 4^a^	100 ± 2	131 ± 6^b^	115 ± 4^c^
BW (g)	179 ± 1	185 ± 4	187 ± 4	364 ± 4	400 ± 12	310 ± 22^d^	511 ± 5	465 ± 17	341 ± 31^b,e^
CCr (ml/min/100 g)	n.d.	n.d.	n.d.	n.d.	n.d.	n.d.	0.72 ± 0.05	0.24 ± 0.03^b^	0.29 ± 0.01^b^
FeP (%)	n.d.	n.d.	n.d.	n.d.	n.d.	n.d.	4.14 ± 0.30	13.13 ± 1.89^b^	0.34 ± 0.08^c^
Urinary Ca (mg/day)	1.92 ± 0.62	2.38 ± 0.38	3.47 ± 0.70	0.75 ± 0.14	0.82 ± 0.15	3.93 ± 0.77^b,c^	0.57 ± 0.15	0.62 ± 0.07	3.95 ± 0.90^b,c^
BUN (mg/dl)	n.d.	n.d.	n.d.	n.d.	n.d.	n.d.	20.3 ± 0.7	86.4 ± 21.7^b^	46.9 ± 3.1
Plasma Ca (mg/dl)	n.d.	n.d.	n.d.	n.d.	n.d.	n.d.	10.6 ± 0.2	11.1 ± 0.1	13.2 ± 0.2^b,e^
Hb (g/dl)	n.d.	n.d.	n.d.	n.d.	n.d.	n.d.	12.9 ± 0.2	11.2 ± 0.8	12.8 ± 0.7
intact PTH (pg/ml)	n.d.	n.d.	n.d.	n.d.	n.d.	n.d.	146.8 ± 21.6	866.7 ± 376.7	5.9 ± 0.8^c^
1,25(OH)_2_ VitD (pg/ml)	n.d.	n.d.	n.d.	n.d.	n.d.	n.d.	132 ± 12.3	160 ± 37.1	405 ± 41.3^b,e^
Ca × P	n.d.	n.d.	n.d.	n.d.	n.d.	n.d.	59.0 ± 3.9	80.1 ± 5.0^b^	33.5 ± 2.2^b,e^

BP, blood pressure; HR, heart rate; BW, body weight; FeP, fractional excretion of phosphate; Hb, hemoglobin; VitD, vitamin D.

^a^P < 0.01 versus control at 4 weeks; ^b^P < 0.01 versus control at 8 weeks; ^c^P < 0.05 versus RK at 8 weeks; ^d^P < 0.01 versus RK at 4 weeks; ^e^P < 0.01 versus RK at 8 weeks.

### Administration of SF ameliorates glomerulosclerosis and tubulointerstitial injury in RK rats

**Figure 2:**
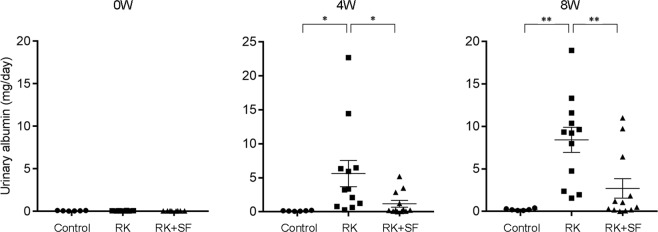
SF ameliorates albuminuria in RK model. Levels of urinary albumin at 0, 4, and 8 weeks in the indicated animals (*n* = 6 for control, *n* = 12 for RK, *n* = 12 for RK + SF groups). Data are expressed as mean ± SEM; *P < 0.05. **P < 0.01.

To evaluate whether SF exerted protective effects on renal injury in RK rats, we measured urinary albumin levels at 0, 4, and 8 weeks. Urinary albumin levels were comparable among groups at baseline. RK rats showed a marked increase in urinary albumin levels at 4 weeks and at 8 weeks (Fig. 2). Compared with RK group, urinary albumin levels were significantly decreased in RK + SF group at both 4 and 8 weeks (Fig. 2).

**Figure 3:**
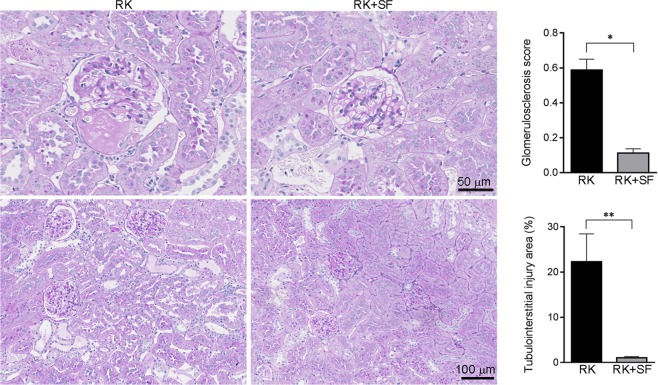
SF ameliorates glomerular and tubulointerstitial injury in RK model. Representative periodic acid-Schiff (PAS)-stained kidney sections from RK and RK + SF rats. Bar graphs show the histological analysis of glomerulosclerosis (*n* = 4 each group) and of tubulointerstitial injury (*n* = 8 each group) in RK and RK + SF groups (see also Methods). Data are expressed as mean ± SEM; *P < 0.05. **P < 0.01.

To determine whether SF alleviated renal injury, we also addressed renal histology in periodic acid-Sciff (PAS)-stained kidney sections. Consistent with previous reports^23,24^, RK rats showed glomerulosclerosis and tubulointerstitial damages including tubular dilatation, tubular atrophy and intraluminal cast (Fig. 3). Semiquantitative evaluation of renal histology demonstrated that SF significantly alleviated the glomerular and tubulointerstitial damages.

### SF confers protection against glomerular podocyte injury in RK model

**Figure 4:**
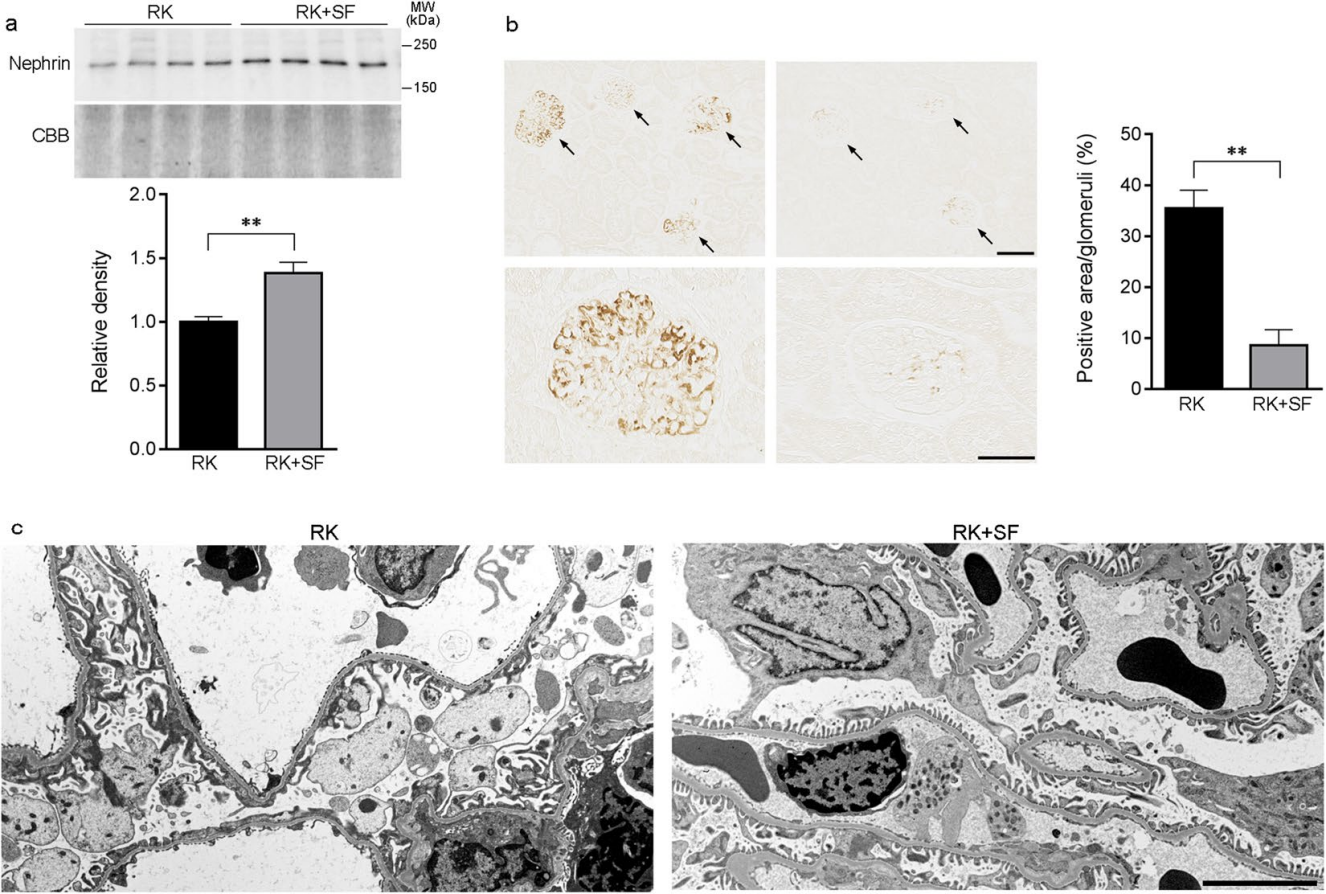
Glomerular podocyte injury in RK rats is attenuated by SF. (**a**) Western blot analysis of nephrin in the plasma membrane-enriched fraction of the kidney in RK and RK + SF rats. Bar graphs show the results of quantitation (*n* = 4 each group). (**b**) Representative micrographs of immunostaining for desmin, a marker for podocyte injury. Bar graphs show the quantitative evaluation of desmin staining in the glomeruli. Bar in the upper panel represents 100 μm. Bar in the lower panel represents 50 μm. (**c**) Transmission electron micrographs of podocyte foot process in the glomeruli of indicated animals. Bar represents 5 μm. Data are expressed as mean ± SEM; *P < 0.05. **P < 0.01.

Existing evidence suggests a role of glomerular podocyte in the development of glomerulosclerosis and the progression of renal dysfunction in CKD^23,25,26^. We next addressed whether SF administration in RK rats was associated with the amelioration of glomerular podocyte injury. Western blot analysis revealed that the expression of the slit diaphragm protein nephrin was significantly higher in RK + SF rats than in RK rats (Fig. 4a)^27^. Conversely, immunohistochemical study revealed that the expression of desmin, a sensitive marker for podocyte injury^28,29^, was significantly attenuated in podocytes in the glomeruli of RK + SF rats (Fig. 4b). Consistent with these findings, a structural analysis using transmission electron microscopy demonstrated that the extensive foot process effacement in RK rats were partially attenuated by SF administration (Fig. 4c). These data indicate that SF attenuates glomerular podocyte injury and are consistent with the previous observation indicating the causal role of phosphate load in the podocyte injury^30^.

### SF alleviates renal inflammation and fibrosis in RK model

**Figure 5:**
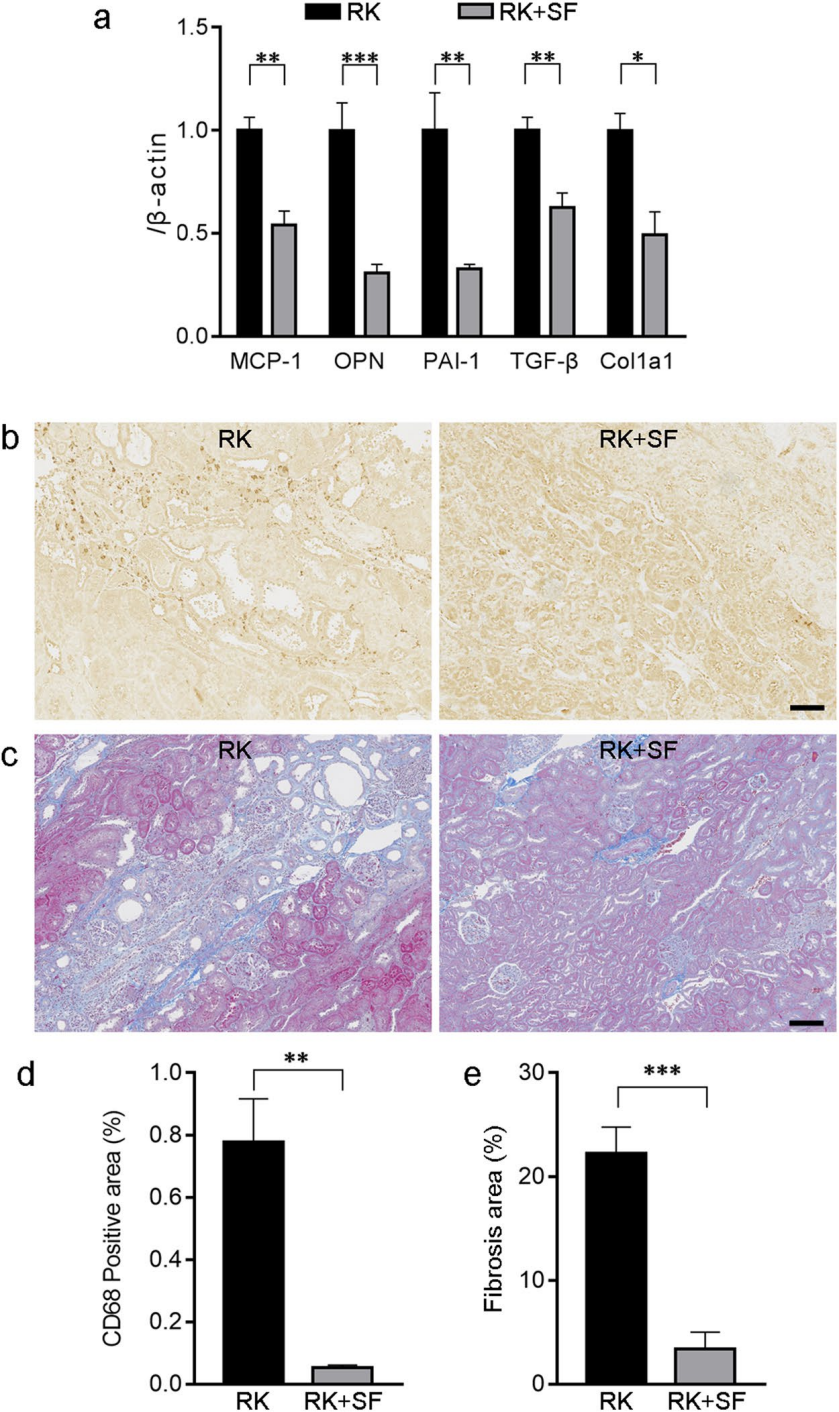
Renoprotective effects of SF is associated with the alleviation of renal inflammation and fibrosis. (**a**) Quantitative analysis of MCP-1, osteopontin (OPN), PAI-1, TGF-β, and α1(I) collagen (Col1a1) gene expression by real time RT-PCR in the kidney of RK and RK+SF rats (n = 8 each group). (**b**) Immunohistochemical staining for ED-1 (CD68) in the kidneys of RK (left) and RK + SF (right) rats. Infiltration of the CD68-positive macrophages (indicated by arrows) in RK rats was ameliorated by SF. Bar represents 100 μm. (**c**) Masson’s trichrome staining of the kidney sections from RK (left) and RK + SF (right) rats. Fibrotic area was reduced by SF administration. Bar represents 100 μm. (**d,e**) Quantitative analysis of the CD68-positive area (**d**) and fibrosis area (**e**) (see also Methods; *n* = 4 each group). Data are expressed as mean ± SEM; *P < 0.05. **P < 0.01. ***P < 0.001.

We next evaluated the possible mechanism for the renoprotective effects observed in RK + SF rats. Previous studies documented the roles of several cytokines that are responsible for renal inflammation and fibrosis in RK model^23,24,31–33^. Therefore, we quantitative analyzed the gene expression of these cytokines in RK and RK + SF rats. Quantitative real time RT-PCR revealed that the expression of pro-inflammatory cytokines including MCP-1, osteopontin, and PAI-1 were significantly decreased in the kidney by SF (Fig. 5a). In addition, the expression of pro-fibrotic molecules TGF-β and α1(I) collagen (Col1a1) was also significantly attenuated in the kidney (Fig. 5a).

To validate the gene expression analysis above, we evaluated the infiltration of macrophage by ED-1 (CD68) staining. Quantitative evaluation revealed that the infiltration of macrophages in the kidney was significantly reduced in SF + RK group (Fig. 5b,d). We also evaluated renal fibrosis by Masson’s trichrome stain, which demonstrated that the fibrotic area was again significantly reduced by SF administration in RK rats (Fig. 5c,e). These data indicate that phosphate binding by SF ameliorates renal injury in RK rats by preventing inflammatory and fibrogenic responses.

### Renoprotective effects of SF are independent of ectopic renal calcification

**Figure 6:**
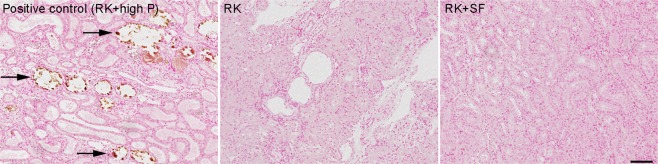
Protective effects of SF in RK rats are independent of CaPi deposition in the kidney. Representative von Kossa-stained kidney sections from RK (middle) and RK + SF rats (right). In the left panel, RK rats fed high (1.2%) phosphate diet served as a positive control for the renal calcification (indicated by arrows). Bar represents 100 μm.

Previous studies reported that the deleterious effects of phosphate in the kidney are mediated by crystal nephropathy resulting from ectopic calcification^11,13–16^. Therefore, we addressed the possible contribution of CaPi deposition in the kidney in our model. For this purpose, kidneys from RK and RK + SF group were stained with von Kossa staining. As a positive control, we used a remnant kidney rat that received 1.2% phosphate diet for 8 weeks. As shown in Fig. 6 (left panel), these rats showed overt intraluminal calcification in the renal tubules. In sharp contrast, we found no evidence for CaPi deposition in RK nor RK + SF rats in the renal parenchyma at 8 weeks (Fig. 6, middle and right panels). These data indicate that the renoprotective effects of SF observed in our study are independent of overt CaPi crystal formation in the renal parenchyma.

### SF reduces the formation of plasma CaPi nanoparticles in RK rats

**Figure 7:**
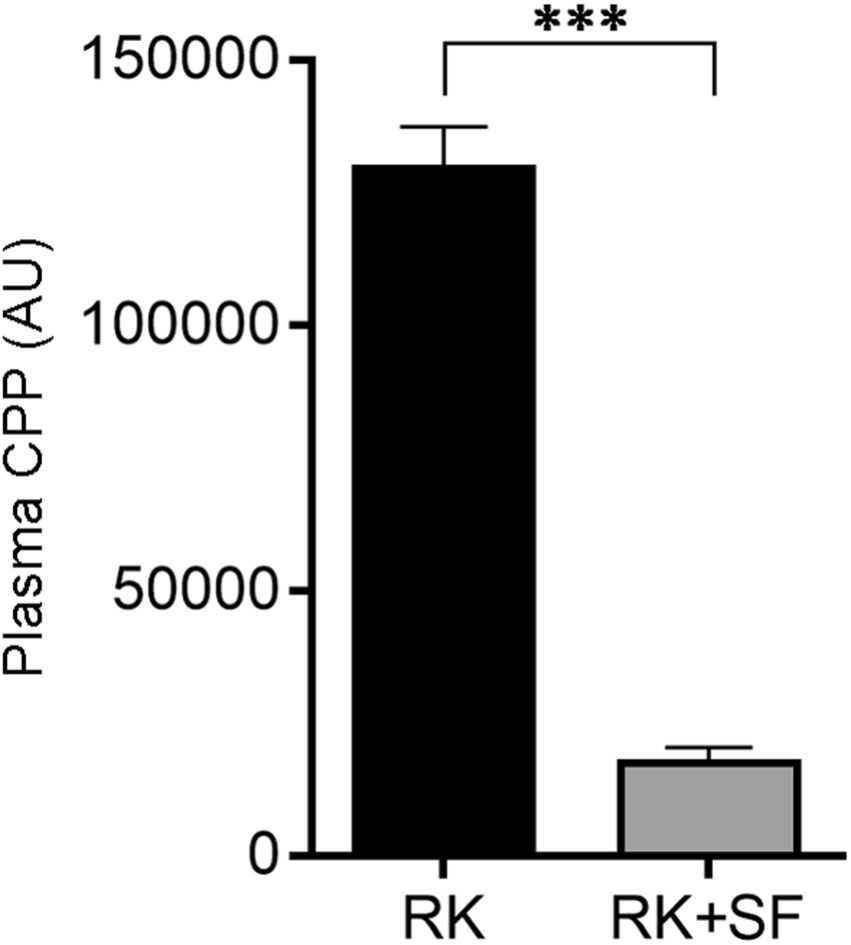
Plasma calciprotein particle levels in RK and RK + SF rats. Plasma levels of calciprotein particle (CPP) were measured in RK and RK + SF rats (*n* = 4 each group). ***P < 0.001.

Next, we evaluated whether SF ameliorated the formation of CaPi nanoparticles in the plasma^22,34^. The results showed that plasma CPP levels were significantly reduced in RK + SF rats compared with RK rats (Fig. 7). These data demonstrate that SF prevented the formation of CaPi nanoparticles in the blood, confirming that the disturbed mineral metabolism in RK model is alleviated by SF.

**Figure 8:**
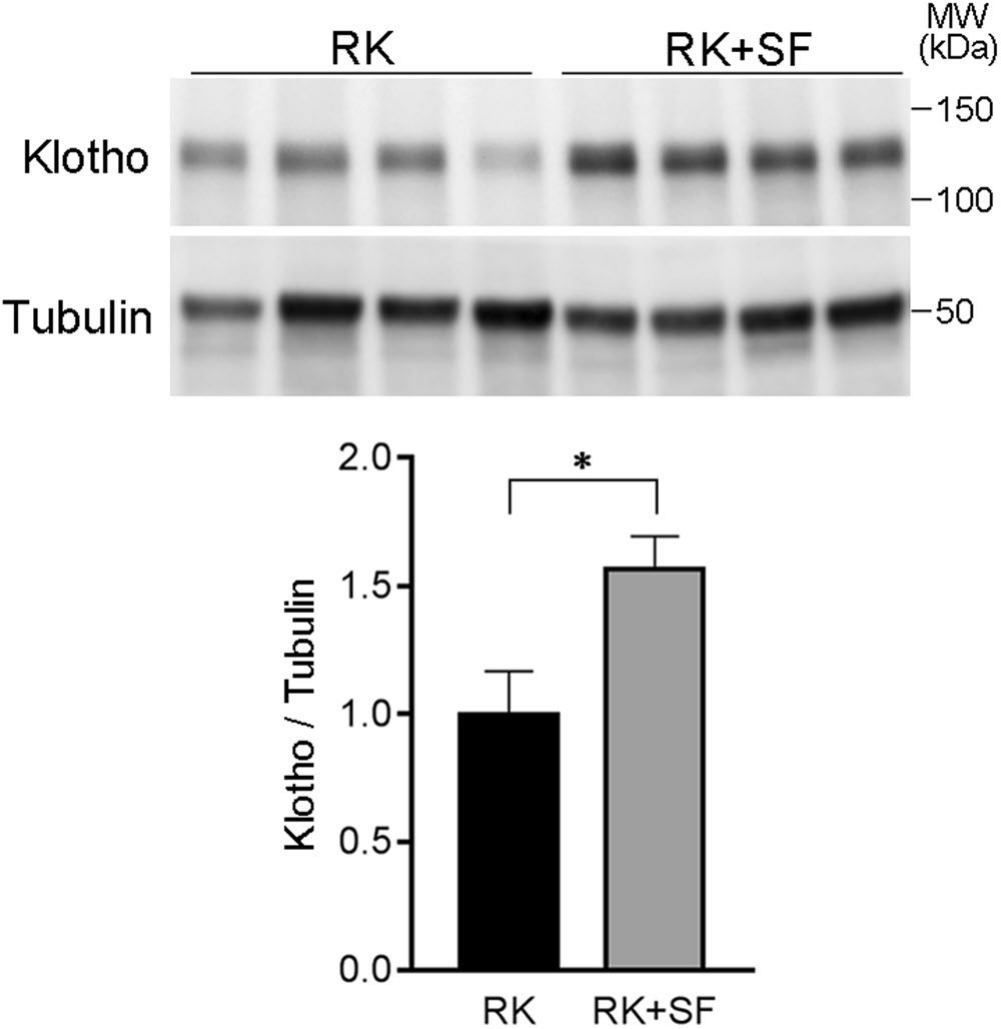
Renal Klotho levels in RK and RK + SF rats. Levels of Klotho were evaluated in the kidney homogenates in RK and RK + SF rats. Bar graphs show the results of quantitation (*n* = 5 each group). *P < 0.05.

Given the evidence that phosphate loading and phosphaturia alter renal Klotho expression levels^35^, we evaluated Klotho abundance in our model. Consistent with these reports, we found that Klotho levels were higher in RK + SF rats than in RK rats (Fig. 8), confirming that phosphate loading to renal tubules reduces Klotho abundance.

### Relationship between urinary albumin excretion and other parameters at 8 week using all rats

In order to obtain insights into the factors contributing to the protective effects of SF observed in this study, the multiple linear regression for urinary albumin excretion at 8 week was performed using other confounders obtained at the same experimental week. Multiple linear regression was conducted in a stepwise manner with inclusion if *F* value ≥ 2.0 and exclusion if *F* value < 2.0. As shown in Table 2, plasma phosphate was first selected as a significant independent variable (model 1) and then removed when urinary phosphate was added (model 3). The final model chose urinary phosphate excretion and CCr as significant independent variables, explaining 81% of urinary albumin levels (model 4). The result indicated that urinary excretion of phosphate rather than plasma phosphate had a stronger impact on urinary albumin excretion, the decline of which was achieved by the administration of SF but not by the decrease in body weight or the change in blood pressure. The association of CCr and urinary albumin excretion was considered to be a simple reflection of the CKD rat model used. There was no multicollinearity because variance inflation factor (VIF) ranged from 1.00 to 6.38 through the analysis. Blood pressure and other parameters such as plasma levels of FGF23, intact PTH, 1,25(OH)_2_ vitamin D and CPP failed to show the significant result.

**Table 2:** Multiple linear regression with urinary albumin excretion regarding other parameters at 8 weeks.

CKD stage	Characteristic	β	*t* value	*P* value	Adjusted *R*^2^	*F* value	*P* value
Model 1	Plasma phosphate	0.32	4.25	<0.001	0.45	18.0	<0.001
Model 2	Plasma phosphate CCr	0.31	5.93	<0.001	0.73	30.1	<0.001
		−2.52	−4.72	<0.001			
Model 3	Plasma phosphate CCr Urine phosphate	0.05	0.43	0.67	0.80	28.5	<0.001
		−3.55	−5.84	<0.001			
		0.30	2.63	0.017			
Model 4	CCr Urine phosphate	−3.71	−7.81	<0.001	0.81	44.6	<0.001
		0.35	7.43	<0.001			

Multiple linear regression analysis for urinary albumin excretion was performed in an incremental inclusion manner using other independent parameters at 8 weeks including body weight, blood pressure, CCr, urinary phosphate, plasma phosphate, plasma FGF23, plasma intact PTH, plasma 1,25(OH)_2_ vitamin D and plasma CPP. Urinary excretion of albumin and phosphate and plasma levels of FGF23, intact PTH, 1,25(OH)_2_ vitamin D and CPP were log-transformed prior to the analysis. Abbreviations. CCr: creatinine clearance corrected for body weight of 100 g; FGF23, fibroblast growth factor 23; CPP, calciprotein particle.

## Discussion

Using the rat remnant kidney model, we here demonstrate that intestinal phosphate binding by sucroferric oxyhydroxide (SF) counteracts against the progression of CKD. SF ameliorated glomerulosclerosis and tubulointerstitial injury. Moreover, SF conferred protection on podocytes, explaining the attenuation of glomerulosclerosis and albuminuria observed in this study. We found that SF attenuated renal inflammation and fibrosis, which likely contributed to the protective effects in RK model. Importantly, these effects are independent of ectopic calcification, because von Kossa staining revealed CaPi deposition in the kidney in neither RK nor RK + SF rats.

We speculate that there are several possibilities that explain the mechanisms whereby the reduced glomerular function and the resultant phosphate accumulation caused renal injury in our model. Firstly, hyperphosphatemia accelerates the growth and precipitation of CaPi crystals, which can contribute to renal dysfunction through tubular obstruction and inflammation^13–15^. The toxic effects of phosphate overloading and of the resultant ectopic calcification are well illustrated by the pathophysiology of phosphate nephropathy, in which phosphate-based cathartic agents induce acute renal dysfunction associated with diffuse and abundant intratubular CaPi deposits^36,37^. The obstruction of renal tubules by CaPi crystals results in cellular necrosis and inflammation followed by tubulointerstitial injury. However, neither macroscopic nor microscopic CaPi deposition was present in the renal parenchyma in our model, suggesting the involvement of alternative mechanisms. Moreover, glomerular structure is usually preserved in acute phosphate nephropathy^36,38^, which does not fully explain the observations of the current study.

Second possibility is that phosphate, by itself or phosphate-containing factors such as CPP, contributed to the renal dysfunction. The pathogenic role of phosphate overload to glomerular podocytes has been previously described^30^. In addition, several studies indicate that CPP can act as bioactive ligands that can significantly modify cellular function^8,19,20^. Kuro-o *et al*. proposed that increased phosphate loading promotes CPP formation, which is cytotoxic to renal tubule cells through the induction of renal inflammation^34^. Consistently, recent evidence indicates that phosphate accumulation can cause kidney injury independently of CaPi deposition in the kidney^38^. Besides phosphate and CPP, humoral factors might also have contributed to the pathogenesis or protective effects. For example, FGF23 has been reported to cause cardiovascular dysfunction^39–41^. Given the evidence that vitamin D can ameliorate renal injury in several rodent models^42,43^, it is also possible that the elevation in vitamin D levels in RK + SF rats contributed to the renoprotective effects of SF rats at least in part. Direct demonstration of the underlying mechanisms whereby the disturbed phosphate metabolism facilitates renal cell injury would need cell culture experiments, which was not addressed in this study.

The mechanisms for the reduction in blood pressure in RK + SF groups at 8 weeks are unclear. Given that the blood pressure levels at 4 weeks (at which time we already observed the difference in albuminuria between RK and RK + SF groups) were similar between the two groups, we infer that phosphate load and mineral stress contributed to the progression of kidney dysfunction in our model via mechanisms other than changes in blood pressure. It is possible that SF protected against vascular dysfunction in this model through their effects on mineral metabolism. This inference is supported by the previous studies suggesting that the disturbed mineral metabolism in CKD contributes to cardiovascular disorder^5,11,12,41^. In addition, there is also evidence that FGF23 regulates blood pressure through the effects on renal salt handling^44^.

The importance of urinary phosphate excretion in relation to urinary albumin excretion might be emphasized in the present study, given that the multiple linear regression analysis chose this parameter as a significant independent parameter. Despite the initial selection of plasma phosphate level as an independent parameter, the significance was overridden when urinary phosphate excretion was added to the model, suggesting the impact on the effect of urinary albumin excretion. We found that fractional excretion of phosphate (FeP) was significantly increased in RK rats, which was attenuated by SF administration, further supporting the pathogenic importance of phosphate loading per nephron. These data are in line with recent studies showing the pathogenic role of phosphaturia in accelerating the decline in kidney function^35^.

A limitation of our study is that the exact mechanisms whereby phosphate binding by SF alleviates renal inflammation and fibrosis remain undetermined. It is also unknown whether SF is protective in other models of kidney injury, such as hypertensive and diabetic kidney diseases. Also, SF administration was associated with the reduction in body weight. The reason for the changes remains unclear; however, we speculate that hypophosphatemia might have delayed the growth in RK + SF group. Although the multivariate analysis excluded the possibility the effect of change in body weight, it is worth evaluating the efficacy of a lower dose of SF with a minimal effect on body weight in the future study. In addition, it must also be noted that RK + SF rats showed over-suppression of PTH and hypercalcemia, the important risk factors for adynamic bone disease and cardiovascular events, respectively^45,46^. In our study, calcium phosphate product was reduced by SF due to the reduction in plasma phosphate levels, which is consistent with the decreased CPP levels and the lack of ectopic calcification. However, the effects of SF on bone tissue were not determined. Despite these limitations, the current study clearly demonstrates that the disturbed mineral metabolism contributes to the progression of renal injury, even when ectopic calcification is not evident. Given our data, we speculate that the dietary phosphate restriction can also have similar protective effects in CKD model. It is also possible that phosphate overload causes renal injury even when plasma phosphate levels are not elevated. Our data indicate that the early intervention against positive phosphate balance may be effective in improving the prognosis of CKD.

## Methods

### Animal studies

Animal procedures were approved by the Teikyo University Ethics Committee for Animal Experiments (#15-027) and were conducted in accordance with the guidelines of the Institute Animal Care and Use Committee of the Teikyo University. Male Sprague Dawley rats at 6 weeks of age were obtained from Sankyo Labo Service (Tokyo, Japan). The rats received the surgical resection of the upper and lower one-thirds of the left kidney. The resected portion of the left kidney was weighed to validate the procedure. One week later, the rats received right uninephrectomy. They were then randomly assigned to either the remnant kidney (RK) or the RK with sucroferric oxyhydroxide (RK + SF) group. RK rats received standard chow (AIN-93G, which contained 0.5% of calcium, 0.3% of phosphorus, and 1 IU/g of vitamin D). In RK + SF group, SF was mixed in the same diet at a dose of 50 mg/g chow. At the indicated periods, urine was collected for 24 hours by using individual metabolic cages. Blood pressure was measured by the tail cuff plethysmography. At 8 weeks, animals were euthanized under anesthesia of inhaled isoflurane. Blood samples were obtained by cardiac puncture. Kidneys were removed, and the renal cortex was dissected, snap-frozen, and stored at −80 °C until use. Urinary albumin levels were measured at SRL. Plasma and urinary levels of electrolyte concentrations were measured ion-selective electrodes (SRL). Hemoglobin levels were measured by iSTAT blood gas analyzer (Abbott). Plasma intact-PTH levels were measured by ELISA (Immutopics). Plasma intact FGF23 levels were measured using ELISA kit (Kainos).

### Histology

For morphological evaluations, transverse sections (1 μm) were stained with periodic acid-Sciff (PAS) reagent, and the degrees of glomerulosclerosis and tubulointerstitial injury were semi-quantitatively analyzed in accordance with previous reports^23,47^. Glomerulosclerosis was defined as disappearance of cellular elements from the tuft, capillary loop collapse and folding of the glomerular basement membrane with accumulation of amorphous material. The grades were 0, 0%; I, 1 to 25%; II, 26 to 50%; III, 51 to 75%; and IV, 76 to 100% of glomeruli involved. The glomerulosclerosis score was calculated as (1 × % grade I) + (2 × % grade II) + (3 × % grade III) + (4 × % grade IV). Tubulointerstitial injury was defined as tubular cast formation, tubular atrophy, or thickening of tubular basement membrane. The areas of the injured tubulointerstitium were calculated digitally using an image analysis program (Aperio ImageScope). Renal fibrosis was analyzed in the kidney sections stained with Masson’s trichrome stain. Renal calcification was evaluated by von Kossa staining. As a positive control for von Kossa staining, RK rats received diet containing 1.2% phosphate for 8 weeks; these rats showed frequent deposition of calcium salt (see Results).

### Immunohistochemistry and quantification

After blocking, cryosections were stained with the indicated primary antibodies and affinity-purified secondary antibodies-conjugated HRP (DAKO, Glostrup, Denmark). Primary antibodies used included antibodies against CD68 (Serotec) and desmin (Dako). Areas positive for CD68 were quantitated using NanoZoomer (Hamamatsu Photonics, Hamamatsu, Japan) and Aperio ImageScope (Leica, Buffalo Grove, IL, USA). For quantification of desmin in the glomeruli, the percentage of positive area was determined as positive pixels per total pixels in a glomerulus using Image Scope software. For each rat, 20 glomeruli were randomly analyzed.

### Transmission electron microscopy

Ultramicrostructure of the glomeruli was observed by transmission electron microscopy. Small pieces of cortex were fixed in 2.5% glutaraldehyde, dehydrated through graded ethanol and propylene oxide, and embedded in Epon 812 using standard procedures. Ultrathin sections were stained with uranyl acetate and with Reynolds lead citrate. The specimens were observed using HITACHI transmission electron microscope H-7650 (Hitachi Science Systems Ltd., Hitachinaka, Japan).

### Western blotting

Western blotting was performed as described previously^48^. Plasma membrane fraction was isolated from total kidneys using plasma membrane isolation kit (Minute, Invent biotechnologies, MN, USA), as described previously^49^. For total cell lysates, kidneys were homogenized with TNE buffer. Equal amounts of protein were mixed with Laemmli sample buffer, boiled for 5 min (or incubated at room temperature for 20 min for membrane proteins), separated on polyacrylamide gel, and transferred to PVDF membrane. The membrane was incubated with primary and peroxidase-conjugated secondary antibodies, followed by imaging using ECL reagents (Perkin Elmer). CBB (for plasma membrane fraction) and tubulin (for total cell lysates) were used to ensure equal loading of different samples. Antibody against nephrin was created and characterized as described previously^50^. Monoclonal antibody against Klotho (KM2119, Trans Genic) is characterized in previous studies^51^. Full-length gels and blots are shown in Supplemental Fig. 1. 

### RNA extraction and Quantitative RT-PCR

RNA was extracted using commercially available kit (Qiagen). TaqMan gene expression assays (Thermo Fisher Scientific) were used for the quantitative RT-PCR analysis.

### Measurement of plasma calciprotein particle

Plasma levels of calciprotein particles (CPP) were measured as described previously^22^.

### Statistical analysis

The data are summarized as mean ± SEM. Unpaired *t* test was used for comparisons between two groups. Non-parametric data were analyzed by Mann-Whitney’s U test. For multiple comparisons, statistical analysis was performed by ANOVA followed by Tukey post hoc tests. Multiple linear regression analysis for urinary albumin excretion in total rats was conducted to examine the effect of other parameters. Prior to the analysis, the following variables were log-transformed due to non-normality of the distribution of the data; urinary albumin excretion, plasma FGF23, plasma intact PTH, plasma 1,25(OH)_2_ vitamin D and plasma CPP. Then, multiple linear regression was conducted in in a stepwise manner with inclusion if *F* value ≥ 2.0 and exclusion if *F* value < 2.0. Multicollinerity was checked by variance inflation factor (VIF). A *P* value < 0.05 was considered statistically significant.

## Supplementary information

Supplemental Figure

## Data Availability

The data sets generated during the current study are available on reasonable request.

## References

[1] Jha V (2013). Chronic kidney disease: global dimension and perspectives. Lancet.

[2] Gansevoort RT (2013). Chronic kidney disease and cardiovascular risk: epidemiology, mechanisms, and prevention. Lancet.

[3] Zoccali C (2011). Phosphate may promote CKD progression and attenuate renoprotective effect of ACE inhibition. J Am Soc Nephrol.

[4] Chang WX (2016). The Impact of Normal Range of Serum Phosphorus on the Incidence of End-Stage Renal Disease by A Propensity Score Analysis. Plos One.

[5] Wolf M (2015). Mineral (Mal)Adaptation to Kidney Disease–Young Investigator Award Address: American Society of Nephrology Kidney Week 2014. Clin J Am Soc Nephrol.

[6] Komaba H, Fukagawa M (2016). Phosphate-a poison for humans?. Kidney Int.

[7] Shimada T (2004). Targeted ablation of Fgf23 demonstrates an essential physiological role of FGF23 in phosphate and vitamin D metabolism. J Clin Invest.

[8] Kuro OM (2011). Phosphate and Klotho. Kidney Int.

[9] Weinman EJ (2007). Parathyroid hormone inhibits renal phosphate transport by phosphorylation of serine 77 of sodium-hydrogen exchanger regulatory factor-1. J Clin Invest.

[10] Streja E (2013). Hyperphosphatemia is a combined function of high serum PTH and high dietary protein intake in dialysis patients. Kidney Int Suppl.

[11] Vervloet M, Cozzolino M (2017). Vascular calcification in chronic kidney disease: different bricks in the wall?. Kidney Int.

[12] Jimbo R (2014). Fibroblast growth factor 23 accelerates phosphate-induced vascular calcification in the absence of Klotho deficiency. Kidney Int.

[13] Haut LL, Alfrey AC, Guggenheim S, Buddington B, Schrier N (1980). Renal toxicity of phosphate in rats. Kidney Int.

[14] Lau K (1989). Phosphate excess and progressive renal failure: the precipitation-calcification hypothesis. Kidney Int.

[15] Aihara K, Byer KJ, Khan SR (2003). Calcium phosphate-induced renal epithelial injury and stone formation: involvement of reactive oxygen species. Kidney Int.

[16] Mulay SR, Anders HJ (2017). Crystal nephropathies: mechanisms of crystal-induced kidney injury. Nat Rev Nephrol.

[17] Hamano T (2010). Fetuin-mineral complex reflects extraosseous calcification stress in CKD. J Am Soc Nephrol.

[18] Kuro-o. M. Klotho and endocrine fibroblast growth factors: marker of chronic kidney disease progression and cardiovascular complications? *Nephrol Dial Transplant* (2018). 10.1093/ndt/gfy126 29800324

[19] Smith ER, Hanssen E, McMahon LP, Holt SG (2013). Fetuin-A-containing calciprotein particles reduce mineral stress in the macrophage. Plos One.

[20] Smith ER, Hewitson TD, Hanssen E, Holt SG (2018). Biochemical transformation of calciprotein particles in uraemia. Bone.

[21] Matsui I (2013). Retention of fetuin-A in renal tubular lumen protects the kidney from nephrocalcinosis in rats. Am J Physiol Renal Physiol.

[22] Miura Y (2018). Identification and quantification of plasma calciprotein particles with distinct physical properties in patients with chronic kidney disease. Sci Rep.

[23] Aldigier JC, Kanjanbuch T, Ma LJ, Brown NJ, Fogo AB (2005). Regression of existing glomerulosclerosis by inhibition of aldosterone. J Am Soc Nephrol.

[24] An WS, Kim HJ, Cho KH, Vaziri ND (2009). Omega-3 fatty acid supplementation attenuates oxidative stress, inflammation, and tubulointerstitial fibrosis in the remnant kidney. Am J Physiol Renal Physiol.

[25] Durvasula RV (2004). Activation of a local tissue angiotensin system in podocytes by mechanical strain. Kidney Int.

[26] Nagata M (2016). Podocyte injury and its consequences. Kidney Int.

[27] Kawachi H, Koike H, Shimizu F (2002). Molecular structure and function of the slit diaphragm: expression of nephrin in proteinuric states and in developing glomeruli. Nephrol Dial Transplant.

[28] Yaoita E, Kawasaki K, Yamamoto T, Kihara I (1990). Variable expression of desmin in rat glomerular epithelial cells. Am J Pathol.

[29] Shibata S, Nagase M, Yoshida S, Kawachi H, Fujita T (2007). Podocyte as the target for aldosterone: roles of oxidative stress and Sgk1. Hypertension.

[30] Sekiguchi S (2011). Phosphate overload induces podocyte injury via type III Na-dependent phosphate transporter. Am J Physiol Renal Physiol.

[31] Yu XQ (2000). Osteopontin expression in progressive renal injury in remnant kidney: role of angiotensin II. Kidney Int.

[32] Boor P (2011). The peroxisome proliferator-activated receptor-alpha agonist, BAY PP1, attenuates renal fibrosis in rats. Kidney Int.

[33] Nakagawa T (2011). Keishibukuryogan reduces renal injury in the early stage of renal failure in the remnant kidney model. Evid Based Complement Anternat Med.

[34] Kuro-o M (2013). Klotho, phosphate and FGF-23 in ageing and disturbed mineral metabolism. Nat Rev Nephrol.

[35] Santamaria R (2018). Increased Phosphaturia Accelerates The Decline in Renal Function: A Search for Mechanisms. Sci Rep.

[36] Desmeules S, Bergeron MJ, Isenring P (2003). Acute phosphate nephropathy and renal failure. N Engl J Med.

[37] Markowitz GS, Stokes MB, Radhakrishnan J, D’Agati VD (2005). Acute phosphate nephropathy following oral sodium phosphate bowel purgative: an underrecognized cause of chronic renal failure. J Am Soc Nephrol.

[38] Yamada Y (2016). Acute Phosphate Nephropathy with Diffuse Tubular Injury Despite Limited Calcium Phosphate Deposition. Intern Med.

[39] Schluter KD, Piper HM (1998). Cardiovascular actions of parathyroid hormone and parathyroid hormonerelated peptide. Cardiovasc Res.

[40] Faul C (2011). FGF23 induces left ventricular hypertrophy. J Clin Invest.

[41] Arnlov J (2013). Serum FGF23 and risk of cardiovascular events in relation to mineral metabolism and cardiovascular pathology. Clin J Am Soc Nephrol.

[42] Matsui I (2009). Active vitamin D and its analogue, 22-oxacalcitriol, ameliorate puromycin aminonucleosideinduced nephrosis in rats. Nephrol Dial Transplant.

[43] Zhang Y, Kong J, Deb DK, Chang A, Li YC (2010). Vitamin D receptor attenuates renal fibrosis by suppressing the renin-angiotensin system. J Am Soc Nephrol.

[44] Andrukhova O (2014). FGF23 regulates renal sodium handling and blood pressure. EMBO Mol Med.

[45] Bolland MJ, Grey A, Avenell A, Gamble GD, Reid IR (2011). Calcium supplements with or without vitamin D and risk of cardiovascular events: reanalysis of the Women’s Health Initiative limited access dataset and meta-analysis. BMJ.

[46] Block GA, Kilpatrick RD, Lowe KA, Wang W, Danese MD (2013). CKD-mineral and bone disorder and risk of death and cardiovascular hospitalization in patients on hemodialysis. Clin J Am Soc Nephrol.

[47] Shibata S (2011). Rac1 GTPase in rodent kidneys is essential for salt-sensitive hypertension via a mineralocorticoid receptor-dependent pathway. J Clin Invest.

[48] Shibata S (2013). Mineralocorticoid receptor phosphorylation regulates ligand binding and renal response to volume depletion and hyperkalemia. Cell Metab.

[49] Xu N (2017). Hypokalemia and Pendrin Induction by Aldosterone. Hypertension.

[50] Kawachi H (2000). Cloning of rat nephrin: expression in developing glomeruli and in proteinuric states. Kidney Int.

[51] Kato Y (2000). Establishment of the anti-Klotho monoclonal antibodies and detection of Klotho protein in kidneys. Biochem Biophys Res Commun.

