# Supplementary material for: Phosphate binding by sucroferric oxyhydroxide ameliorates renal injury in the remnant kidney model
Source: Sci Rep. 2019 Feb 11;9:1732. doi: 10.1038/s41598-018-38389-3 (PMC6370755; doi:10.1038/s41598-018-38389-3)

Phosphate binding by sucroferric oxyhydroxide ameliorates renal injury in the remnant kidney model

Content type: Original article

Yoshikazu Nemoto[1)], Takanori Kumagai[1)], Ken-ichi Ishizawa[1)], Yutaka Miura[2)], Takeshi Shiraishi[1)], Chikayuki Morimoto[1)], Kazuhiro Sakai[1)], Hiroki Omizo[1)], Osamu Yamazaki[1)], Yoshifuru Tamura[1)], Yoshihide Fujigaki[1)], Hiroshi Kawachi[3)], Makoto Kuro-o[2)], Shunya Uchida[1)], Shigeru Shibata[1), 4)]

[1)]Division of Nephrology, Department of Internal Medicine, Teikyo University School of Medicine, Tokyo, Japan. [2)]Division of Anti-aging Medicine, Center for Molecular Medicine, Jichi Medical University, Tokyo, Japan. [3)]Department of Cell Biology, Kidney Research Center, Niigata University Graduate School of Medical and Dental Sciences, Niigata, Japan. [4)]Division of Clinical Epigenetics, Research Center for Advanced Science and Technology, The University of Tokyo, Tokyo, Japan.

Address correspondence to: Shigeru Shibata, MD., Ph.D., Division of Nephrology, Department of Internal medicine, Teikyo University School of Medicine, 2-11-1 Kaga, Itabashi-ku, Tokyo 173-8605, Japan. Phone: +81-3-3964-1211; e-mail: shigeru.shibata@med.teikyo-u.ac.jp.

Supplemental Figure 1.

Full-length images of the gels and blots used in Figure 4a and Figure 8.

Nephrin (Fig 4a)

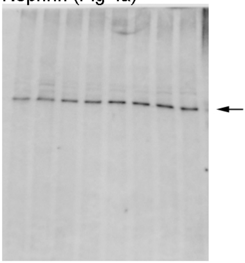

CBB (Fig 4a)

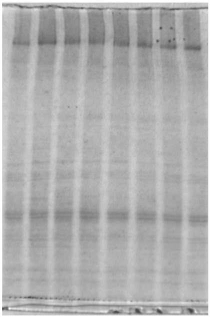

Klotho (Fig 8)

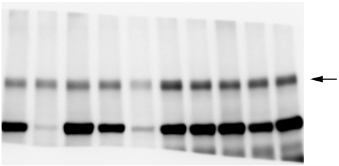

Tubulin (Fig 8)

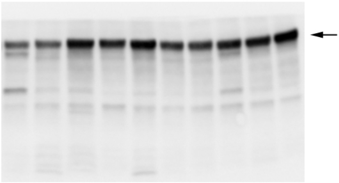

Supplement: Supplementary file 1 — Supplemental Figure [file 41598_2018_38389_MOESM1_ESM.pdf]
